# Neuromuscular and balance adaptations following basketball-specific training programs based on combined drop jump and multidirectional repeated sprint versus multidirectional plyometric training

**DOI:** 10.1371/journal.pone.0283026

**Published:** 2023-03-15

**Authors:** Seifeddine Brini, Daniel Boullosa, Julio Calleja-González, Rodrigo Ramirez-Campillo, Hadi Nobari, Carlo Castagna, Filipe Manuel Clemente, Luca Paolo Ardigò

**Affiliations:** 1 Research Unit, Sportive Performance and Physical Rehabilitation, University of Jendouba, Kef, Tunisia; 2 Integrated Institute of Health, Federal University of Mato Grosso do Sul, Campo Grande, Brazil; 3 College of Healthcare Sciences, James Cook University, Townsville, Australia; 4 Faculty of Physical Activity and Sports Sciences, Universidad de León, León, Spain; 5 Physical Education and Sport Department, Faculty of Education and Sport, University of the Basque Country (UPV/EHU), Vitoria-Gasteiz, Spain; 6 Faculty of Kinesiology, University of Zagreb, Zagreb, Croatia; 7 Department of Physical Activity Sciences, Universidad de Los Lagos, Santiago, Chile; 8 Exercise and Rehabilitation Sciences Institute, School of Physical Therapy, Faculty of Rehabilitation Sciences, Universidad Andres Bello, Santiago, Chile; 9 Department of Exercise Physiology, Faculty of Educational Sciences and Psychology, University of Mohaghegh Ardabili, Ardabil, Iran; 10 Department of Motor Performance, Faculty of Physical Education and Mountain Sports, Transilvania University of Braşov, Braşov, Romania; 11 Faculty of Sport Sciences, University of Extremadura, Cáceres, Spain; 12 School of Sports and Exercise Science, University of Rome Tor Vergata, Rome, Italy; 13 Fitness Training and Biomechanics Laboratory, Italian Football Federation (FIGC), Technical Department, Florence, Italy; 14 Escola Superior Desporto e Lazer, Instituto Politécnico de Viana do Castelo, Viana do Castelo, Portugal; 15 Research Center in Sports Performance, Recreation, Innovation and Technology (SPRINT), Melgaço, Portugal; 16 Instituto de Telecomunicações, Delegação da Covilhã, Lisboa, Portugal; 17 Department of Teacher Education, NLA University College, Oslo, Norway; Universidade da Beira Interior; Research Center in Sports Sciences, Health Sciences and Human Development (CIDESD), PORTUGAL

## Abstract

Multidirectional jumping and repeated sprint ability are crucial performance factors in basketball. The main aim of this investigation was to examine the neuromuscular performance and body balance adaptations following basketball-specific combined training programs based on drop jump and multidirectional repeated sprint versus multidirectional plyometric training. Forty-two professional basketball male players participated in the current investigation and were randomly assigned to three groups: a combined group (COMB; n = 14), a multidirectional jump group (MJG; n = 14) and an active control group (CON; n = 14). The COMB and the MJG groups completed the 8-week training programs with two weekly sessions while the CON continued their usual training. The static and dynamic balance tests, the repeated sprint ability test (IRSA_5COD_), the T–change of direction (CoD) test, the vertical jump tests, the five time-jump test (FJT) were performed by participants before and after the intervention period. The results showed a significant main effect of time with remarkable improvements at the end of the intervention (*P* < 0.001, effect size *small*/*moderate*) except the physiological parameters for IRSA_5COD_. Only, significant group × time interactions for body balance, T-CoD test, IRSA_5COD_ (total time and best time), and jump tests were found (*P* < 0.001, effect size from *trivial* to *moderate*). Bonferroni corrected *post-hoc* tests revealed significantly greater improvement in favor of COMB compared to MJG for body balance, CoD and IRSA_5COD_ (*P* < 0.005, effect size *small*/*moderate*). Otherwise, no significant differences between COMB and MJG concerning jump performances were found. Combined drop jump and multidirectional repeated sprint training program lead to significantly better neuromuscular performance, body balance and CoD in professional basketball players when compared with an usual training.

## Introduction

Repetitive multidirectional movements such as sprinting and jumping are crucial performance factors and critical physical demands in basketball [1–3]. In the same context, previous studies have reported the importance of sprints with change of direction (CoD) among basketball players [4, 5]. Additionally, the nature of basketball games requires players to jump (i.e., lay-up, jump-shot, rebound, block shot, and so on [6]) and their jumps are generally made multidirectional (i.e., vertical, horizontal, bilateral, unilateral, etc. [7]) and according to the situations imposed by the opponent, the coaches’ strategies (attack or defense), and the position of the ball on the court [7]. In the same context, previous studies recommended using multidirectional plyometric and sprinting training programs to improve muscular strength and power, decrease injuries and optimize performances [8–10]. Based on the importance of the multidirectional sprinting and jumping in basketball and by following the principle of specificity and transfer of training [11], it will be interesting to implement a training program based on these two qualities, which mainly aim to improve them specifically and other bio-motor abilities in general in a combined or a single mode strategy [12, 13].

Previous investigation reported the positive impact of CoD in stressing dynamic balance by challenging the ability to maintain or return the center of gravity over the base of support [14]. In the same context, it has been shown that plyometric training position as a dynamic form of resistance training with a rapid stretch-shortening cycle (SSC), may stimulate body balance and help players to control their body by involving both vertical and horizontal displacements of the individual’s center of gravity [15–17]. Recently, Brini et al. [12] showed that an 8-week combined training program based on drop jump and multidirectional repeated sprint training contributed to significantly better body balance and CoD performance in professional basketball players. Therefore, those authors suggested that the combination of strength, power, and CoD ability is the best specific conditioning method to win a duel in basketball [12].

On the other hand, a recent review suggested that horizontal plyometric training is practical as vertical plyometric training in improving performance in actions with a vertical direction, but it is superior in improving performance in horizontally oriented skills [9]. In the same context, several studies reported the importance of using the combination of vertical and horizontal jumps as an advantageous option to maximize gains in jump, sprinting, and CoD performance [18, 19]. Additionally, previous studies recommended carrying out future interventions using multidirectional plyometric training, including horizontal and vertical jumps, as an optimal training strategy [18, 19]. Finally, although the positive effects of the combined training (drop jump and multidirectional repeated sprints) and multidirectional plyometric training in the literature, their impacts in the field of professional basketball and to what extent each type of training improves certain specific physical and physiological parameters are as yet unknown and need more futures investigations.

To the best of author’s knowledge, the present investigation is the first to explore neuromuscular performance and balance adaptations following basketball-specific training programs based on drop jump and multidirectional repeated sprint versus multidirectional plyometric training in professional basketball male players. Therefore, the present study aimed to examine the effect of specific combined training programs based on drop jump and multidirectional repeated sprint versus multidirectional plyometric training in professional basketball male players. Considering previous literature [12, 13, 18–20], we hypothesized that both training programs would significantly enhance neuromuscular performances and body balance.

## Materials and methods

### Study design and setting

The present study assessed adaptations following combined training programs based on drop jump and multidirectional repeated sprint versus multidirectional plyometric training using a parallel group randomized study design that included pre- and post-testing and two training interventions. Overall, the experimental intervention–pre-testing, training and post-testing–lasted eleven weeks (October to December 2021) during the competitive season. The detailed weekly training programs of the three groups are presented in Table 1. Body balance, repeated sprint ability performance and lower limbs power were tested before and after the eight-week training intervention. The random allocation sequence, enrolled, and assigned participants to the intervention groups was generated by two independent researchers [21]. Participants were assigned using Research Randomizer (http://www.randomizer.org) to a combined training group (COMB), a multidirectional jump group (MJG) and an active control group (CON).

**Table 1:** Weekly training program during the experimental period for intervention and control groups.

Days	Training program for intervention groups (COMB and MJG)	Training program for the active control group (CON)
**Monday**	• Warm-up, 15 min • Specific basketball fundamental training, 15 min • Moderate intensity mid-range and 3-point shot exercises, 20 min • Free throw shooting, 10 min • Technical/Tactical training, 25 min	• Warm-up, 15 min • Specific basketball fundamental training, 15 min • Moderate intensity mid-range and 3-point shot exercises, 20 min • Free throw shooting, 10 min • Technical/Tactical training, 25 min
**Tuesday**	• Warm-up, 15 min • The training intervention (COMB or MJG) 20 min • Free throw shooting, 10 min • Moderate intensity mid-range and 3-point shot exercises, 20 min • Technical/Tactical training, 25 min	• Warm-up, 15 min • The regular strength training (Lower body) 20 min • Free throw shooting, 10 min • Moderate intensity mid-range and 3-point shot exercises, 20 min • Technical/Tactical training, 25 min
**Wednesday**	• Warm-up, 15 min • Specific basketball fundamental training, 10 min • Ball drill transition training, 15 min • The regular strength training (Upper body) 20 min • Three-point shot exercises, 15 min • Tactical training, 15 min	• Warm-up, 15 min • Specific basketball fundamental training, 10 min • Ball drill transition training, 15 min • The regular strength training (Upper body) 20 min • Three-point shot exercises, 15 min • Tactical training, 15 min
**Thursday**	• Warm-up, 15 min • The training intervention (COMB or MJG) 20 min • Free throw shooting, 10 min • Moderate intensity mid-range and 3-point shot exercises, 20 min • Technical/Tactical training, 25 min	• Warm-up, 15 min • The regular strength training (Lower body) 20 min • Free throw shooting, 10 min • Moderate intensity mid-range and 3-point shot exercises, 20 min • Technical/Tactical training, 25 min
**Friday**	• Warm-up, 15 min • Free throw shooting, 15 min • Low-intensity 3-point shooting exercises, 30 min • Tactical training, 15 min • Free throw shooting, 10 min	• Warm-up, 15 min • Free throw shooting, 15 min • Low-intensity 3-point shooting exercises, 30 min • Tactical training, 15min • Free throw shooting, 10 min
**Saturday**	• Match	• Match
**Sunday**	• Recovery	• Recovery

## Participants

Forty-two professional (elite) basketball male players participated in the present study (Table 2). Participants were from different 3 teams of the same level in the first division Tunisian championship. Their training experience and weekly exposure were 12.5 ± 2.9 years and ≈ 9 hours, respectively.

**Table 2:** Anthropometric characteristics of the participants.

Groups	Age (years)	Height (cm)	Body Mass (kg)
**COMB (n = 14)**	25.1±2.3	196.4±6.0	85.2±5.7
**MJG (n = 14)**	24.8±1.7	195.8±5.4	86.4±4.5
**CON (n = 14)**	26.0±2.1	196.2±3.5	85.4±4.2

Data are reported as mean and standard deviation. COMB: Combined group; MJG: Multidirectional jump group; CON: Control group; BMI: Body mass index.

**Fig 1:**
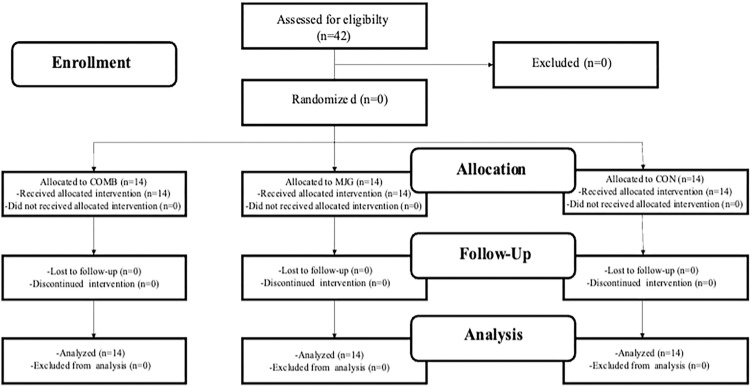
Flow chart of the progress through the phases of the study according to the CONSORT statements.

An a priori power analysis [22] with an assumed Type I error of 0.01 and a Type II error rate of 0.10 (90% statistical power) was conducted for results [23] in the Y-balance test as a proxy of dynamic balance and revealed that 42 persons would be sufficient to observe a medium group × test interaction effect. Players were randomly assigned to combined training group (COMB; n = 14), a multidirectional jump group (MJG; n = 14) and an active control group (CON; n = 14). The inclusion criteria in the present investigation were that participants had no history of musculoskeletal, neurological or orthopedic disorders that might affect their ability to perform physical fitness tests and a high intensity effort. The flow chart of the study design was described in Fig 1.

This study was approved by the ethics committee of the local university (approval no. 12/2017). The experimental protocol was conducted according to the latest version of the Declaration of Helsinki. All participants provided their written informed consent before study participation.

### Procedures

Before the start of the experimental period, participants performed some familiarization sessions to understand all the tests protocols. During the test session participants were instructed to wear the same sports equipments and the same time of the testing was respected all over the experimental period to minimize any effects of diurnal variations [24]. Throughout the experimental period, the MJG, COMB and CON groups completed the same training volume (~ 90 minutes per session) in which training sessions started with a 15-minute warm-up followed by technical and tactical drills based on basic basketball movements and a simulated game at the end of every session [7] (Table 1).

In addition, the participants performed four testing sessions (each one following a 5-min jogging warm-up) before (T1) and after (T2) the intervention distributed as follows: the first testing session was devoted to vertical jump tests, the second testing session was devoted to CoD and five time-jump (FJT), the third testing session was devoted to the balance tests and the fourth testing session was devoted to the repeated sprint test. The testing sessions followed at least a 48-hour withdrawal from strenuous physical activity and were separated each other by at least 48 hours. The tests during the same session were performed randomly.

### Training program interventions

**Fig 2:**
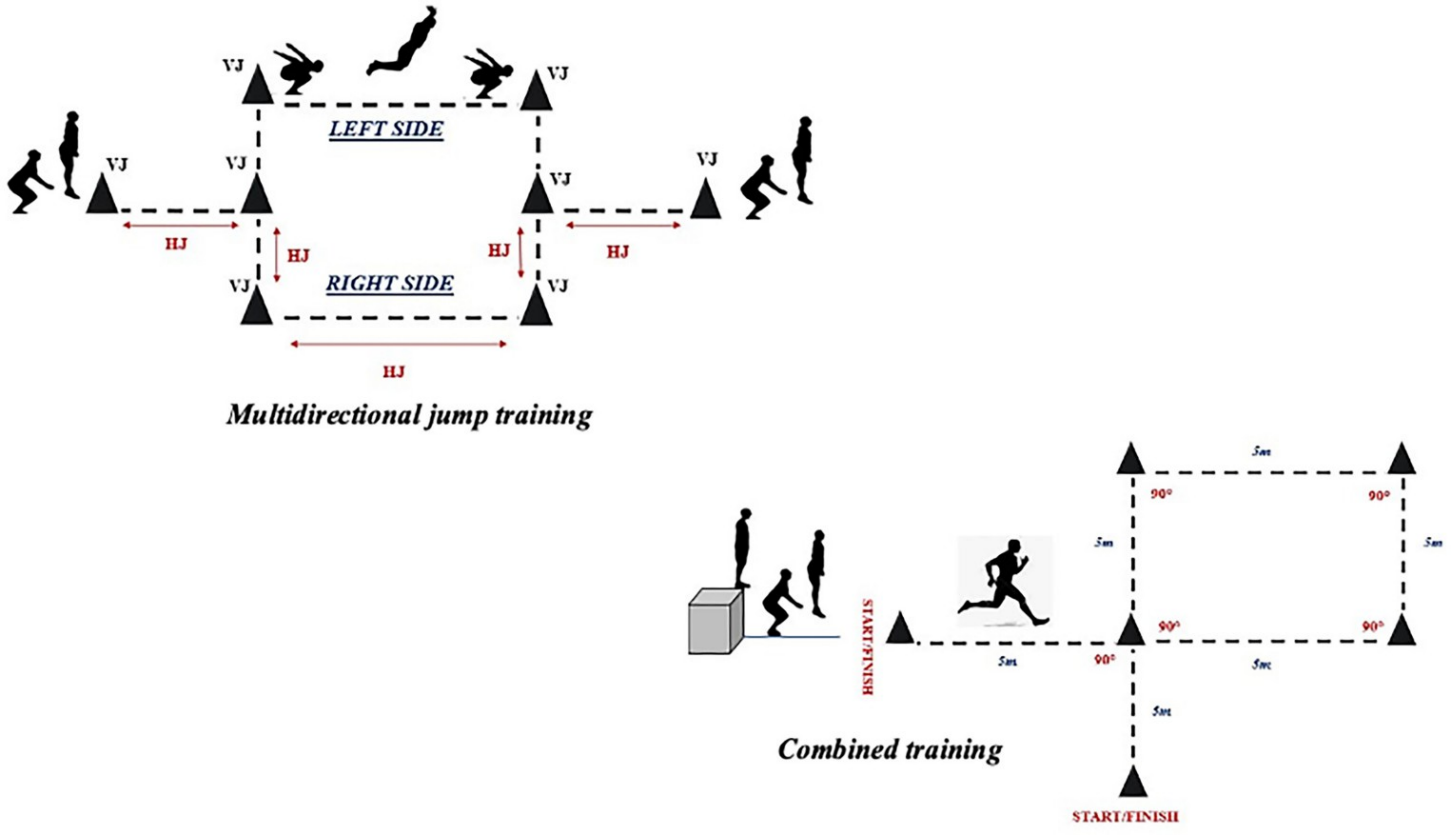
The training protocol for the MJG and COMB.

During the experimental period, participants were encouraged to give their maximal effort. The training weeks, sets, repetitions and duration were matched between groups in order to ensure an equal training volume and were controlled by a professional strength and conditioning coach. In addition, each training session started with a 15 min warm-up and finished with 10 min cool down. The training intervention for COMB consisted of 3 sets of 8 repetitions (DJ +30 m sprint with CoD) and the number of repetitions increased to 10 during the second month. The recovery process was 20 seconds between the repetitions and a 4 min rest between sets (Fig 2). Concerning the MJG, the training intervention consisted of 3 sets of 10 repetitions (5 vertical jumps + 5 horizontal jumps) and the number of repetitions increased to 12 during the second month. The recovery process was 40 seconds and 3 minutes recovery between the repetitions and sets respectively. The jumps were performed in the following order (first set: jumping with both legs; second set: jumping only with the right leg; third set: jumping only with the left leg) (Fig 2).

### Training load monitoring

**Fig 3:**
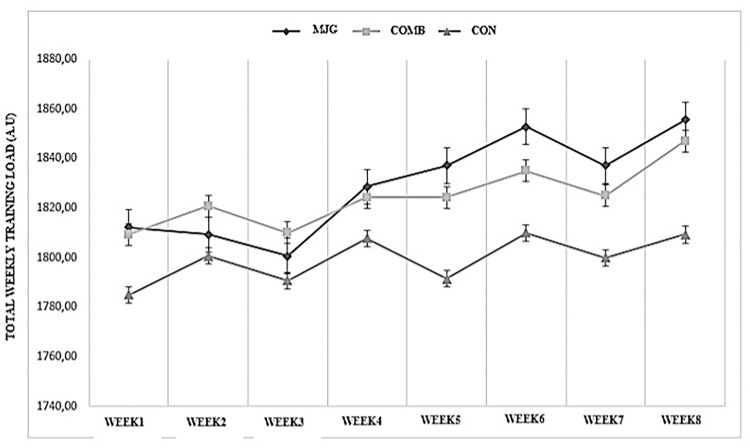
Total weekly training load for the MJG, COMB and CON.

The training load during the present investigation was measured according to the methods described by Foster et al. [25]. Participants were asked to rate the global intensity of the entire workout session using the category ratio-10 (RPE) scale 30-minute following each session. Daily training intensity was created by multiplying the training duration (minutes) by the session RPE and the weekly training load was determined by summing the daily training loads for each athlete during each week (Fig 3).

### Measures

#### Anthropometrics and maximal oxygen consumption

For the anthropometrics, the collected measurements were: Body mass (kg) was measured with an electronic scale (Pharo, 200 Analytic, Germany), body mass index (BMI) and height (m) measured with a portable stadiometer (Seca, Maresten, UK). Concerning the estimation of maximal oxygen consumption (VO_2Max_), Participants performed the 20-m shuttle run test before the beginning of the experimental intervention and the VO_2Max_ was calculated according to the equation of Léger and Gadoury [26].

#### Balance tests

In order to assess the participants’ static and dynamic body balance, the Stork and the Y-balance balance tests were used respectively [27, 28]. The Stork balance test protocol consisted of standing with both hands on your hips then the participants place the sole of the non-standing foot against the inside knee of the other leg and raise the heel from the floor The assistant starts the stopwatch as soon as your heel is off the floor [27]. The test was terminated when the heel of the supporting leg touched the ground, or the foot moved away from the kneecap [27]. Concerning the Y-balance test, participants were instructed to maintain single-leg balance while reaching as far as possible with the other leg in three different directions (anterior, posteromedial and posterolateral) performed on each leg following the previously described protocol [28]. The composite score for each participant was calculated as the average maximum reach across the three directions [29]. For Both balance tests participants performed three trials interspaced by 2 min recovery trials and the best of trials was used for data analysis.

#### T–change of direction test

The CoD T-test was performed with four cones forming a T and an electronic timing system (Brower Timing Systems, Salt Lake City, UT, USA) was used to record times following the previously described protocol [30]. This adapted test requires sprinting, lateral shuffling, and backpedaling with 4 directional changes representing the typical movements of basketball [30]. Two trials interspaced by 2 min were completed and the fastest trial was taken for further analysis.

#### Intensive repeated sprint ability test

The IRSA_5COD_ test is a valid specific test in basketball players [30]. Participants performed 10×30-m sprints with three CoD following a T shape separated by 30 s of recovery [31]. The measured parameters during this test were: total time (TT), best time (BT). The time for each attempt was recorded with photocells (Brower timing system, Salt Lake City, UT, USA), fatigue index (FI) was calculated using the Fitzsimons et al. [32] formula: (100 × (TT / (BT × 10)) − 100), the heart rate recorded using a cardio-frequency monitor (Polar Electory, Kempte, Finland). At the end of the test, the rating of perceived exertion (RPE) was assessed [33] in addition to the maximal blood lactate concentration which was measured at the third minute [34] using a portable lactate analyzer (Arkray Lactate Pro LT-1710 Kyoto, Japan) [35].

#### Vertical jump tests

In order to assess vertical jump, the countermovement (CMJ) and the squat jumps (SJ) tests were performed according to previously described protocols using an optoelectrical system (Opto-Jump Microgate, Italy) in which jump height was calculated according to the following equation: jump height = 1 / 8 × g × t^2^ [36, 37]. To assess interlimb asymmetry, one leg drop jump was performed using a 30-cm box. The asymmetry index using the following formula: ((Highest performing limb–Lowest performing limb) / Highest performing limb) × 100 [38]. All tests were performed three times, interspaced by 2 min. The best of trials was used for further data treatment.

#### Five-time jump test

The FJT test was performed according to the previously described protocol [39]. Participants were instructed to keep their feet in parallel position at the beginning of the test and after the fifth jump performance and the test performance was recorded in meters (m) to the nearest cm. The test was in performed two trials interspaced by 2 min and the best trial was used for further analyses.

### Statistical analyses

All the data were presented as mean and standard deviation (SD). The Shapiro-Wilk test identified all variables as normally distributed. The baseline between group differences was computed using one-way ANOVA.

The training effects were evaluated using a 3 (groups: COMB, MJG and CON) × 2 (time: Pre-test, Post-test) mixed model ANOVA. If a statistically significant interaction effect was found, Bonferroni corrected *post-hoc* tests were calculated.

Additionally, effect sizes (ES) were determined from ANOVA output by converting partial eta-squared to Cohen’s *d*. In addition, within-group ES were computed using the following equation: ES = (mean post–mean pre) / SD [40]. Following Hopkins et al. [41], ES were considered *trivial* (<0.2), *small* (0.2 to <0.6), *moderate* (0.6 to <1.2), *large* (1.2 to <2.0) and *very large* (2.0 to 4.0). Additionally, intraclass correlation coefficients (ICC) and coefficients of variation (CV) were computed to assess relative and absolute test-retest reliability (Table 3). ICCs were classified as ICC < 0.50 weak, 0.50 to 0.79 *moderate* and ≥ 0.80 *strong*. The level of significance was set at *P* < 0.05. All statistical analyses were computed using SPSS for Windows, version 20.0 (SPSS Inc., Chicago).

**Table 3:** Intraclass correlation coefficients (ICCs) for relative reliability and coefficients of variation for absolute reliability of the applied physical fitness tests.

Measures			ICC	95% CI	% CV
**SBT**	**R**		0.87	0.80–0.94	3.5
	**L**		0.88	0.81–0.92	3.3
**YBT**	**R**	***Ant***	0.97	0.85–0.98	5.1
		***Post/Md***	0.98	0.84–0.98	5.5
		***Post/Lat***	0.97	0.84–0.99	5.2
	**L**	***Ant***	0.98	0.85–0.98	5.3
		***Post/Md***	0.96	0.87–0.97	5.1
		***Post/Lat***	0.98	0.86–0.99	5.4
**T-test**			0.97	0.89–0.98	3.1
**IRSA**_**5COD**_	**TT**		0.98	0.90–0.98	2.6
	**BT**		0.96	0.87–0.98	2.5
**CMJ**			0.98	0.91–0.99	3.2
**SJ**			0.98	0.93–0.98	3.7
**DJ**	**R**		0.95	0.89–0.98	4.3
	**L**		0.96	0.86–0.97	4.3
**FJT**			0.97	0.87–0.98	3.4

ICC—intraclass correlation coefficient; CI—confidence interval; CV—coefficient of variation (%). IRSA5COD, Repeated sprint ability test with five CoDs.

## Results

All over the intervention period participants completed the different protocols with no major injuries and according to the previously described methodology. Adherence rates were 98.1% for COMB, and 98.3% for MJG. The average playing time per game was 26.3 ± 1.5 min for COMB and 26.1 ± 1.3 for MJG. No statistically significant between-group differences were observed for these measures. In addition, no significant between-group baseline differences were found for any of the analysed parameters (Tables 4 and 5).

**Table 4:** Intensive repeated sprint ability test performances and physiological parameters determined before (pre-test) and after (post-test) the training program.

Groups										*P*-values (effect size)		
Variables	COMB (n = 14)			MJG (n = 14)			CON (n = 14)			Time	Group	Group × Time
	Pre Test	Post Test	Δ%	Pre Test	Post Test	Δ%	Pre Test	Post Test	Δ%			
**TT (s)**	83.56±0.31	82.92±0.27	-0.77±0.12	83.41±0.23	83.04±0.40	-0.45±0.41	83.10±0.11	83.35±0.08	-0.06±0.09	0.000(0.87)	0.176(0.13)	0.000(0.71)
**BT (s)**	8.24±0.08	8.07±0.04	-2.03±0.65	8.18±0.05	8.10±0.03	-0.91±0.55	8.17±0.10	8.16±0.08	-0.09±0.39	0.000(0.88)	0.603(0.03)	0.000(0.80)
**FI (%)**	1.46±0.87	2.77±0.41	123.47±89.87	1.97±0.65	2.43±0.56	43.28±65.49	2.13±1.25	2.15±1.03	7.85±38.68	0.000(0.65)	0.935(0.003)	0.000(0.54)
**HR (beat/min)**	187.10±1.30	187.08±1.33	0.02±0.10	185.81±1.49	186.13±1.41	0.18±0.32	187.06±0.90	187.12±0.87	0.03±0.10	0.042(0.28)	0.024(0.26)	0.119 (0.16)
**[Lac] (mmol/l)**	7.13±0.51	7.11±0.48	-0.05±1.68	7.03±0.46	7.09±0.70	0.81±1.71	7.27± 0.67	7.29± 0.64	0.14±1.31	0.165(0.14)	0.694(0.03)	0.383(0.07)
**RPE**	6.07±0.73	5.86± 0.66	-2.82±11.13	6.14±0.76	5.78±0.70	-5.20±12.02	6.29± 0.73	6.21±0.58	0.14±14.47	0.082(0.21)	0.332(0.08)	0.936 (0.03)

Data are reported as means and standard deviations. TT: total time; BT: best time; FI: fatigue index; HR: heart rate; [Lac]: Lactate concentration; RPE: rating of perceived exertion.

**Table 5:** Body balance, change-of-directions and Jump performances determined before (pre-test) and after (post-test) training program.

Groups												*P*-values (effect size)		
Variables			COMB (n = 14)			MJG (n = 14)			CON (n = 14)			Time	Group	Group × Time
			Pre Test	Post Test	Δ%	Pre Test	Post Test	Δ%	Pre Test	Post Test	Δ%			
**SBT (s)**	**R**		19.06±1.05	23.11±0.90	21.67±8.49	19.56±1.11	21.93±1.92	12.20±8.56	19.48±0.54	19.78±0.77	1.55±3.07	0.000(0.92)	0.004(0.41)	0.000(0.66)
	**L**		19.21±0.82	23.08±1.15	20.46±8.58	19.39±1.23	21.60±1.61	11.38±4.36	19.25±0.94	19.44±1.37	0.99±4.77	0.000(0.88)	0.002(0.46)	0.000(0.76)
**YBT (cm)**	**R**	***Ant***	88.93±3.22	96.93±2.92	9.03±1.58	88±2.91	90.07±3.97	2.35±2.61	88.07±3.50	87.21±5.90	-0.87±6.71	0.000(0.63)	0.001(0.45)	0.000(0.60)
		***Post/Md***	98.21±2.23	109±3.26	10.99±2.44	96.50±4.60	100.64±3.32	4.39±2.59	97.07±2.84	97.14±3.54	0.06±1.63	0.000(0.94)	0.000(0.60)	0.000(0.87)
		***Post/Lat***	58.64±1.15	64.86±1.51	10.63±2.85	59.43±2.38	63.29±2.49	6.51±1.92	58.21±1.19	58.64±1.45	0.75±2.24	0.000(0.94)	0.000(0.60)	0.000(0.84)
	**L**	***Ant***	89.21±3.26	97.21±2.72	9.02±2.38	88.28±2.64	92.35±1.98	4.65±1.62	88.86±2.18	89.07±2.20	0.25±1.44	0.000(0.97)	0.001(0.42)	0.000(0.85)
		***Post/Md***	100.93±2.73	110.93±3.25	9.92±1.95	99.78±2.19	102.79±2.72	3.02±2.10	100.43±1.78	100.79±1.97	0.36±0.89	0.000(0.96)	0.000(0.67)	0.000(0.88)
		***Post/Lat***	58.42±2.40	64.64±1.82	10.74±3.61	58.50±1.29	61.71±2.02	5.54±4.08	58.57±1.74	59.57±1.45	1.73±1.64	0.000(0.91)	0.001(0.49)	0.000(0.69)
**T-test (s)**			6.65±0.07	6.48±0.05	-2.48±0.69	6.67±0.07	6.60±0.06	-1.06±0.78	6.66±0.07	6.65±0.04	-0.24±0.85	0.000(0.90)	0.002(0.38)	0.000(0.66)
**SJ (cm)**			36.79±2.15	39.78±2.19	8.22±2.93	36.86±1.99	39.43±2.87	6.93±4.02	37.71±1.94	37.86±2.03	0.44±3.82	0.000(0.88)	0.768(0.01)	0.000(0.55)
**CMJ (cm)**			38.57±4.70	42.29±4.21	9.97±3.82	38.64±5.67	42.50±5.71	10.19±2.72	37.92±2.46	38.14±2.68	0.64±4.59	0.000(0.94)	0.253(0.10)	0.000(0.69)
**DJ (cm)**	**R**		14,21±1.31	16.21±1.42	14.26±5.56	15±2.11	17.07±1.86	14.50±8.14	14.71±1.73	14.86±1.61	1.36±7.29	0.000(0.85)	0.131(0.15)	0.000(0.58)
	**L**		14.36±1.33	15.79±1.19	10.23±5.26	15.14±2.66	16.93±2.34	12.69±7.60	15±1.52	15.36±1.39	2.85±8.86	0.000(0.78)	0.285(0.09)	0.005(0.37)
	**ASI**		9.53±5.62	7.62±5.15	26.79±158.69	13.20±6.07	11.60±7.37	-0.65±63.74	12.41±5.88	10.11±6.24	-2.42±71.85	0.08(0.0.24)	0.16(0.13)	0.53(0.05)
**FJT (m)**			7.97±0.55	8.09±0.54	1.56±1.09	7.98±0.39	8.21±0.40	2.92±0.84	8.16±0.60	8.18±0.58	0.21±0.43	0.000(0.92)	0.773(0.02)	0.000(0.72)

Data are reported as means and standard deviations. SBT: Stork balance test; YBT: Y-balance test; R: right leg; L: left leg; ***Ant***: anterior; ***Post/Md***: postero-medial; ***Post/Lat***: T-test: CoD T test; SJ: squad jump test, CMJ: countermovement jump test; DJ: single leg drop jump test; ASI: asymmetry index; FJT: five time-jump test.

### Reliability

Reliability measures ICCs ranged from 0.87 to 0.99 and CV ranged from 2.2 to 5.5 for all tests and was presented in Table 3.

### Main effects

All measures displayed significant main effect, (*small*/*moderate*) magnitude (effect size) improvements for time (post-test > pre-test) (Tables 4 and 5) except for physiological parameters for (IRSA_5COD_). Significant main effects for the group were evident with the two groups except for jump performances (Table 5). With each of these measures, the control group differed significantly from the MJG and COMB groups.

### Interactions

#### Body balance

Significant group × time was observed for SBT test performance on both legs (right leg: *P* < 0.001, ES = 0.66, *moderate*; left leg: *P* < 0.001, ES = 0.76, *moderate*). Bonferroni corrected *post-hoc* test for the right leg revealed significant pre-to-post improvements for MJG and COMB with a better improvement in favor COMB (12.20%, *P* < 0.001, ES = 0.44, *small*; and 23.11%, *P* < 0.001, ES = 0.39, *small*; respectively).

Significant group × time interaction was observed for YBT test performance for the right leg support (anterior: *P* < 0.001, ES = 0.60, *moderate*; posteromedial: *P* < 0.001, ES = 0.87, *moderate*; posterolateral: *P* < 0.001, ES = 0.84, *moderate*). Bonferroni corrected *post-hoc* test revealed significant pre-to-post improvements for MJG and COMB with a better improvement in favor of COMB (MJG: anterior: 2.35%, *P* = 0.006, ES = 0.63, *moderate*; posteromedial: 4.39%, *P* < 0.001, ES = 0.60, *moderate*; posterolateral: 6.51%, *P* < 0.001, ES = 0.31, *small*); (COMB: anterior: 9.03%, *P* < 0.001, ES = 0.35, *small*; posteromedial: 10.99%, *P* < 0.001, ES = 0.66, *moderate*; posterolateral: 10.63%, *P* < 0.001, ES = 0.45, *small*); respectively]. For the left support leg, we found similar results (anterior: *P* < 0.001, ES = 0.88, *moderate*; posteromedial: *P* < 0.001, ES = 0.69, *moderate*; posterolateral: *P* < 0.001, ES = 0.66, *moderate*). Bonferroni corrected *post-hoc* test revealed significant pre-to-post improvements for MJG and COMB with a better improvement in favor COMB (MJG: anterior: 4.65%, *P* < 0.001, ES = 0.35, *small*; posteromedial: 3.02%, *P* < 0.001, ES = 0.57, *small*; posterolateral: 5.54%, *P* = 0.001, ES = 0.65, *moderate*; COMB: anterior: 9.02%, *P* < 0.001, ES = 0.53, *small*; posteromedial: 9.92%, *P* < 0.001, ES = 0.53, *small*; posterolateral: 10.74%, *P* < 0.001, ES = 0.52, *small*; respectively).

#### Change of direction

Significant group × time interaction was observed for the T-test (*P* < 0.001, ES = 0.55, *small*). Bonferroni corrected *post-hoc* test revealed significant pre-to-post improvements for MJG and COMB with a better improvement in favor of COMB (-1.06%; *P* < 0.001, ES = 0.01, *trivial*; -2.48%, *P* < 0.001, ES = 0.01, *trivial*; respectively).

#### Intensive repeated sprint ability test

Significant group × time interaction was observed for TT (*P* < 0.001, ES = 0.71, *moderate*). Bonferroni corrected *post-hoc* test revealed significant pre-to-post improvements for MJG and COMB with a better improvement in favor of COMB (-0.47%, *p* < 0.001, ES = 0.01, *trivial*; -0.77%, *P* < 0.001, ES = 0.01, *trivial*; respectively).

Significant group × time interaction was observed for BT (*P* < 0.001, ES = 0.80, *moderate*). Bonferroni corrected *post-hoc* test revealed significant pre-to-post improvements for MJG and COMB with a better improvement in favor of COMB (-0.79%, *p* < 0.001, ES = 0.09, *trivial*; -2.03% *P* < 0.001, ES = 0.03, *trivial*; respectively).

#### Vertical and horizontal jump

Significant group × time interaction was observed for CMJ (*P* < 0.001, ES = 0.65, *moderate*). Bonferroni corrected *post-hoc* test revealed significant pre-to-post improvements for MJG and COMB (10.19%; *P* < 0.001, ES = 0.23, *small*; 9.97%, *P* < 0.001, ES = 0.32, *small*; respectively).

Significant group × time interaction was observed for SJ (*P* < 0.001, ES = 0.55, *small*). Bonferroni corrected *post-hoc* test revealed significant pre-to-post improvements for MJG and COMB (12.24%, *P* < 0.001, ES = 0.41, *small*; 6.93%, *P* < 0.001, ES = 0.38, *small*; 8.22%, *P* < 0.001, ES = 0.28, *small*; respectively).

Significant group × time was observed for DJ test performance on both legs (right leg, *P* < 0.001, ES = 0.58, *small*; left leg, *P* = 0.005, ES = 0.37, *small*). Bonferroni corrected *post-hoc* test for the right leg revealed significant pre-to-post improvements for MJG and COMB (14.50%, *P* < 0.001, ES = 0.30, *small*; 14.26%, *P* < 0.001, ES = 0.21, *small*; respectively).

Significant group × time interaction was observed for FJT (*P* < 0.001, ES = 0.72, *moderate*). Bonferroni corrected *post-hoc* test revealed significant pre-to-post improvements for MJG and COMB (3.04%, *P* < 0.001, ES = 0.02, *trivial*; 1.56%, *P* < 0.001, ES = 0.02, *trivial*; respectively).

## Discussion

The main aim of the present investigation was to explore the neuromuscular and body balance adaptations following basketball-specific combined training programs based on drop jump and multidirectional repeated sprint versus multidirectional plyometric training. In general, our findings showed that the two training interventions significantly improved all selected bio-motor parameters compared to the CON. Additionally, a greater improvement was recorded in favor of COMB compared to MJG for body balance, CoD and IRSA_5COD_. No significant differences between COMB and MJG concerning jump performances and lower limb power. Therefore, the data of the present study partially confirmed our hypothesis.

During the present investigation, both training interventions lead to a significantly better body balance, IRSA_5COD_ performances (TT and BT), CoD and jump performances. The significant improvement may explain our finding of the body balance in motor coordination [42] and the improved neuromuscular control of lower limb muscle after the combined training [43]. In this context, our findings corroborate the data of several previous studies [13, 27]. In the present study, improvements in balance performances following multidirectional plyometric training were in line with previous studies incorporating vertical and horizontal jumping training exercises [9, 44, 45]. The improvement in balance performance may be related to improved co-contraction of lower body muscles [46] and/or to changes in proprioception and neuromuscular control [47]. This improvement in postural control for the experimental group could reflect either improvement in motor output of the lower extremity muscles [48] and/or changes in proprioception and neuromuscular control [47].

Regarding the IRSA_5COD_ test, the significant improvement of the TT and BT may be explained by the enhancements in explosive power through improvements in motor unit synchronization, SSC efficiency, or musculotendinous stiffness following the combined training program [49, 50]. Concerning the significant improvement of the IRSA_5COD_ performances following the multidirectional plyometric training, our results may be explained by the change in explosive performance after a plyometric training program, which may contribute to improvement during repeated sprint tests with CoD [49]. Moreover, several earlier plyometric studies have shown that this type of exercise can enhance sprinting performance in basketball and soccer players [16, 51]. Additionally, it was reported that the eccentric strength gained following the plyometric training is an important determinant of deceleration, especially during CoD actions [52]. In the same context, it was shown that higher inertia accumulated in the braking phase during plyometric training may have contributed to increases in eccentric workload and, therefore, more considerable strength improvements [53].

Moreover, a previous study has explained the improvements in CoD performance with the interaction of several neuromuscular adaptations (i.e., higher efficiency of SSC) and muscle activation strategies that promote improved inter- and intra-muscular coordination [54]. Otherwise, Miller et al. [55] reported that 6-week in-season multidirectional plyometric training improved performance of the CoD and explained these gains by the increases in muscular power and movement efficiency, which are important in team sports generally and basketball more specifically. In addition, the present specific multidirectional jump design unclouding some 90° angles (right/left) may help and improve CoD performances. Furthermore, improved balance would contribute to improved CoD capacity and perhaps even jumping ability as the take-off angle might be more optimal with better balance [56].

Concerning jump performance, our results followed previous studies that reported a significant improvement following combined and multidirectional plyometric training [27, 57, 58]. Improved jump performance due to plyometric training may be partially attributable to improved motor recruitment, the elastic benefits to the SSC and/or muscle typology shifts [54]. This suggests that the significant improvement in vertical jump height reflects a possible improvement in lower limb strength.

Finally, by comparing the two training interventions, the present study’s findings showed a significantly better improvement recorded in favor of COMB compared to MJG for body balance, CoD and RSA. Concerning body balance, our results may be explained mainly by the nature of the multidirectional training design and the combined mode based on CoDs protocols. In this context, several investigations reported that additional changes in directions activities lead to a greater balance and body control [59, 60]. In the same context, Jones et al. [59] reported that high speed with CoD imposes frequent perturbations upon postural control. Additionally, our results revealed better CoD and IRSA_5COD_ in favor of COMB compared to the MJG, which was somewhat expected since the combined mode was designed and extracted from the IRSA_5COD_ test protocol firstly (by including the same CoD angles and sprint distances). Secondly, the combination of speed, jumping and CoD in the combined mode seems to have a great and specific impact in high-intensity actions better than MJG.

### Limitations

Despite its great novelty, our study has some limitations that warrant further investigations. The present investigation explored these two training methods only in professional basketball male players. Maybe it will be interesting to explore other competitive levels and age groups. One other weakness of the present study is that we did not measure muscle strength measures but have assessed proxies to strength as vertical jumping. Regarding this last’s assessment, we calculated only jump height, which is not that sensitive to detect changes of performance featuring this exercise. Overall, future studies are encouraged to explore different strength measures by using more specific equipment and tests.

## Conclusions

The combined drop jumps and multidirectional repeated sprint training program led to a significantly better neuromuscular performance, body balance and CoD in professional basketball male players. Multidirectional plyometric training may be and effective and appealing training option for basketball trainers and coaches.

### Practical applications

The findings of the present study showed that regular strength training is not enough to increase some specific key performance determinants in professional basketball players during an 8-week period. Thus, we suggest that more oriented specific training programs based on combined multidirectional sprint and plyometric training will be a better strategy to optimize training time and to foster basketball-specific performances.

## Supporting information

S1 Data (XLSX)

## List of abbreviations

DJ: drop jump
MJG: multidirectional jump group
COMB: combined group
CON: active control group
STB: Stork test
YBT: Y-Balance Test
IRSA_5COD_: the repeated sprint ability test with CoD
TT: total time
BT: best time
FI: fatigue index
HR: heart rate
RPE: rating of perceived exertion
SJ: squat jump
CMJ: countermovement jump
FJT: five time-jump test
CoD: change of direction
